# Supplementary material for: Cell-cycle-gated feedback control mediates desensitization to interferon stimulation
Source: eLife. 2020 Sep 18;9:e58825. doi: 10.7554/eLife.58825 (PMC7500952; doi:10.7554/eLife.58825)
Supplement: Supplementary file 1. [file elife-58825-supp1.docx]

**Table S1. Primers used in this study**

| Primer names | Sequence |
| --- | --- |
| ACTB_gRNA5_F | CACCGGCCGCGCTCGTCGTCGACAA |
| ACTB_gRNA5_R | AAACTTGTCGACGACGAGCGCGGCC |
| ACTB_PAM_F | CGCTCGTCGTTGATAACGGCTCCGGCATGTGCAAG |
| ACTB_PAM_R | GCCGTTATCAACGACGAGCGCGGCGATATCATCATCC |
| STAT1_gRNA1_F | CACCGCCTAGAAACACAGGATGTGA |
| STAT1_gRNA1_R | AAACTCACATCCTGTGTTTCTAGGC |
| STAT1_PAM_F | CTCTGTTGCTTCACATCCTGTGTTTCTAGGGAAATGAAAGAAAGGCC |
| STAT1_PAM_R | GGATGTGAAGCAACAGAGTAGCAGGAGGGAATCACAGATGAGAAGG |
| IRF9_gRNA2_F | CACCGCTCAGCTACTTCCGCCTGCG |
| IRF9_gRNA2_R | AAACCGCAGGCGGAAGTAGCTGAGC |
| IRF9_PAM_F | CCTTGGGACAGAGTATCCCCCGCAGGCGCAAGC |
| IRF9_PAM_R | AATTGCTTGCGCCTGCGGGGGATACTCTGTCCCAAGGGTAC |
| USP18_gRNA3_F | CACCGGCAAATCTGTCAGTCCATCC |
| USP18_gRNA3_R | AAACGGATGGACTGACAGATTTGCC |
| USP18 PAM_F | CATCCTCGCTGAGTCCTCGCAGTCCCCGGC |
| USP18 PAM_R | CTCAGCGAGGATGGACTGACAGATTTGCCTCAGGAGCC |
| shRNA_USP18 | TAAAAAAGGAGAAGCATTGTTTTCAAATCTCTTGAATTTGAAAACAATGCTTCTCCTGGG |
| shRNA_neg | TAAAAACAGTCGCGTTTGCGACTGGTCTCTTGAACCAGTCGCAAACGCGACTGGGG |
| shRNA_SOCS1 | CCGGGCACTTCCGCACATTCCGTTCCTCGAGGAACGGAATGTGCGGAAGTGCTTTTTG |
