# Supplementary material for: Cell-cycle-gated feedback control mediates desensitization to interferon stimulation
Source: eLife. 2020 Sep 18;9:e58825. doi: 10.7554/eLife.58825 (PMC7500952; doi:10.7554/eLife.58825)
Supplement: Supplementary file 2. [file elife-58825-supp2.docx]

**Table S2. Plasmids constructed in this study**

| Plasmid names | Descriptions |
| --- | --- |
| NHB0234 | *ACTB*-gRNA5 in pSpCas9(BB)-P2A-Puro |
| NHB0250 | Nuclear marker donor: 1kb_UP-NLS-iRFPx2-P2A-1kb_Down in pUC19 |
| NHB0186 | STAT1 gRNA1 in pSpCas9(BB)-P2A-Puro |
| NHB0235 | STAT1 donor: 1.5kb_Up-mCherry-1.5kb_Down in pUC19 |
| NHB0434 | IRF9 gRNA2 in pSpCas9(BB)-P2A-Puro |
| NHB0443 | IRF9 Donor: 1kb_Up-mCitrine-P2A-1kb_Down in pUC19 |
| NHB0503 | shRNA for USP18 in pSuperRetro-puro |
| NHB0504 | shRNA for negative control in pSuperRetro-puro |
| NHB0616 | shRNA for SOCS1 in pLenti-puro |
| NHB0636 | *USP18* gRNA3 in pSpCas9(BB)-P2A-Puro |
| NHB0647 | 1.5kb_Up-NLS-mCerulean-P2A-1.5kb_Down in pUC19 |
| NHB0670 | DHB-mCherry in pLenti-puro |
| NHB0817 | pCMV-mCherry-Gem1-P2A-mCitrine-Cdt1 in pLenti-puro |
