# Supplementary material for: Cell-cycle-gated feedback control mediates desensitization to interferon stimulation
Source: eLife. 2020 Sep 18;9:e58825. doi: 10.7554/eLife.58825 (PMC7500952; doi:10.7554/eLife.58825)
Supplement: Supplementary file 3. [file elife-58825-supp3.docx]

**Table S3. Best-fit parameter values of the model.**

| $\tau$ | Delay in USP18 upregulation (hr) | 8 |
| --- | --- | --- |
| $k_{1}$ | Positive feedback strength (a.u.hr^-1^) | 33.33 |
| $k_{2}$ | Positive feedback saturation constant (a.u.) | 274.29 |
| $k_{3}$ | Negative feedback saturation constant (a.u.) | 549.99 |
| $k_{4}$ | IRF9 production rate (a.u.hr^-1^) | 1.89 |
| $k_{5}$ | USP18 production rate (a.u.hr^-1^) | 86.87 |
| $IRF9_{0}$ | IRF9 basal level (a.u.) | 45 |
| $USP{18}_{0}$ | USP18 basal level (a.u.) | 0 |
